# HTLV-1 epigenetic modification of the FoxP3 TSDR in HAM/TSP decreases the functional proliferative suppression of Tregs

**DOI:** 10.1186/1742-4690-11-S1-O34

**Published:** 2014-01-07

**Authors:** Monique R Anderson, Yoshimi Akahata, Raya Massoud, Nyater Ngouth, Unsong Oh, Steven Jacobson

**Affiliations:** 1 Neuroimmunology Branch, Viral Immunological Section, National Institutes for Neurological Diseases and Stroke, National Institutes of Health, Bethesda, MD, USA; 2 Howard Hughes Medical Institute- National Institutes of Health Research Scholars Program, Howard Hughes Medical Institute. Chevy Chase, MD, USA; 3 Department of Neurology, Virginia Commenwealth University. Richmond, VA, USA

## 

HTLV-1 is a human retrovirus that is associated with adult T-cell leukemia/ lymphoma (ATLL) as well as the neuroinflammatory disorder HTLV-1 associated myelopathy/ tropical spastic paraparesis (HAM/TSP). In these patients, HTLV-1 is primarily found in the CD4+CD25+ T cell subset (Regulatory T cells or Tregs), the cells that are responsible for peripheral immune tolerance and which are known to be dysfunctional in HAM/TSP. However, due to the inherent inflammatory component of HAM/TSP, markers normally used to characterize T regs, such as CD25, FoxP3, and CTLA4 are problematic in differentiating Tregs. Recent evidence has shown that FoxP3 expression and function is determined epigenetically, specifically through DNA methylation in the Treg-specific methylation region (TSDR). To more precisely characterize Treg cells, we analyzed the methylation status of specific CpGs in the TSDR in PBMCs, CD4+ T cells, and CD4+CD25+ T cells from normal healthy donors (NDs) and HAM/TSP patients. We demonstrated that there is decreased demethylation in PBMCs and CD4+CD25+ T cells from HAM/TSP patients as compared to NDs, despite the increased CD4+CD25+ frequency in HAM/TSP. Further, decreased TSDR demethylation correlates with decreased functional suppression in Treg cells of HAM/TSP patients. Additionally, increased HTLV-1 tax expression in PBMC culture correlates with this decrease in FoxP3 TSDR demethylation. Overall, we suggest that HTLV-1 infection decreases Treg functional suppressive capacity in HAM/TSP through epigenetic modification within the FoxP3 locus and that this dysregulation of Treg function may contribute to HAM/TSP disease pathogenesis.

